# Fully automated real-time PCR for *EGFR* testing in non-small cell lung carcinoma

**DOI:** 10.1007/s00428-018-2486-y

**Published:** 2018-11-23

**Authors:** Richard Colling, Hollie Bancroft, Gerald Langman, Elizabeth Soilleux

**Affiliations:** 1 Nuffield Division of Clinical Laboratory Sciences, John Radcliffe Hospital, University of Oxford, Oxford, OX3 9DU UK; 2 Department of Cellular Pathology, John Radcliffe Hospital, Oxford University Hospitals NHS Trust, Oxford, OX3 9DU UK; 3 Department of Histopathology, Birmingham Heartlands Hospital, Heart of England NHS Foundation Trust, Bordesley Green Estate, Birmingham, B9 5SS UK; 4 Division of Cellular and Molecular Pathology, Addenbrooke’s Hospital, Lab Block Level 3, Box 231, Cambridge, CB2 0QQ UK

**Keywords:** Lung cancer, NSCLC, EGFR, Molecular pathology

## Abstract

Molecular testing for mutations in the *EGFR* gene is commonplace for patients with non-small cell lung cancer (NSCLC). These patients are often very sick and management decisions need to be made urgently. In many cases, the results of molecular testing are needed the same day, in order to start targeted therapy and allow maximum benefit for patients. The Idylla™ *EGFR* Mutation Test offers rapid results within three hours of requesting. This study aimed to assess the concordance of Idylla™ *EGFR* Mutation Test results with current standard tests. Forty formalin-fixed, paraffin-embedded NSCLC tumour cases (20 *EGFR* mutant and *EGFR* 20 wild type) were analysed by the Idylla™ *EGFR* Mutation Test (CE-IVD) and compared with PCR and NGS methodologies. The overall concordance between Idylla™ and standard testing was 92.5% (95% CI 80.14% to 97.42%) and the specificity of Idylla™ was 100% (95% CI 83.89% to 100%). The sensitivity was affected by loss of tumour content in tissue blocks in a small number of NGS cases; however, comparing Idylla™ with PCR alone, there was 100% concordance (95% CI 89.85% to 100%). The Idylla™ *EGFR* Mutation Test shows comparative accuracy to routine PCR testing for the most common *EGFR* mutations in NSCLC. The Idylla™ also offers significantly reduced turn-around times compared with existing modalities and therefore the platform would be a useful addition to many molecular diagnostics units.

## Introduction

Lung cancer is the third most common cancer in the UK with around 46,000 cases diagnosed each year. The prognosis for these patients is extremely poor with the overall 10-year survival at only 5% [1]. Non-small cell lung carcinoma (NSCLC) accounts for 85% of these tumours [2] and while surgery and chemoradiotherapy remain the conventional management for most patients, newer targeted therapies have shown great promise in specific subgroups. Around 16% of NSCLC patients have somatic mutations in the epidermal growth factor receptor (*EGFR*) gene and these patients show a greater response to EGFR-tyrosine kinase inhibitors (EGFR-TKi), such as gefitinib, than they do to traditional chemotherapies. Conversely, patients without such mutations respond better to conventional drugs. In the UK, guidance from the National Institute for Health and Care Excellence (NICE) recommends that *EGFR* testing is therefore carried out for all adults with previously untreated, locally advanced or metastatic NSCLC in order to inform clinical management in these patients. A number of next-generation sequencing (NGS) and polymerase chain reaction (PCR) tests for detecting (*EGFR*) mutations are approved by NICE; however, these all generally involve long preparation, require significant staff training, result in a turn-around time of several days (usually due to the need to batch cases), require large tissue volumes and invariably incur high cost [3–5].

The Idylla™ *EGFR* Mutation Test is a novel test to detect *EGFR* mutations in lung cancer, covering all the clinically relevant mutations in exons 18 to 21 (Table 1). The test is a single-use disposable cartridge that can carry out automated PCR on a single section of formalin-fixed paraffin-embedded (FFPE) tissue containing as little as 10% tumour cells. This requires minimal skill and equipment and can achieve an on-demand result within three hours from the time of the pathologist requesting the test [9]. There have been a number of publications to date showing the high diagnostic accuracy of the Idylla™ System for mutations in *BRAF* and *KRAS* in various tissues, but fewer publications exist for *EGFR* testing with the platform [10–16]. Recent evidence demonstrates high concordance of the prototype (non-CE marked, research use only) Idylla™ *EGFR* Mutation Test with conventional methods [17–20]. This study evaluated the new CE-IVD approved Idylla™ *EGFR* Mutation Test with the main aim to verify the previous validations of the RUO test in a small cohort of patients who have undergone routine PCR testing. We also included a small set of NGS-tested samples to get an indication of how the Idylla would fair against such sequencing assays that are becoming popular and have very low limits of detection.

**Table 1:** Details of the available *EGFR* assay on the Idylla system compared with the commonly used NGS (Ion Torrent) platform. Turn-around times and detection limits (analytical sensitivity) are given as quoted by the manufacturers. The Ion PGM (NGS) panel is that described on the Thermo Fisher website. The manufacturer explains that the theoretical turn-around time (sample to result) for this panel can be as low as a single day or 24 h—however in practice with sample batching and routine laboratory working hours, the results often take several days [6]

Gene Target	Idylla [7]	Ion PGM (NGS) [8]
*EGFR*	Idylla *EGFR* Mutation Test [9] Coverage: Exon 18 point mutations (G719A/C/S), exon 19 deletion (Del9, Del12, Del15, Del18, Del21, Del24), exon 20 point mutations (T790 M, S768I) and insertions (insG, insASV9, insASV11, insSVD, insH), exon 21 point mutations (L858R, L861Q) Detection limit: ‘≤ 5% for most prevalent *EGFR* mutations’ Turn-around time: 2 h (approx.)	AmpliSeq Cancer Hotspot Panel v2 [6] Coverage^†^: Detection limit: 98% detection rate for 5% variant frequency at positions with average sequencing coverage from × 1000 to × 4000 Turn-around time: Single day

*Terminating codon notation

^†^Coverage given is for the codon changes that are likely to be relevant in CRC

## Materials and methods

Ethical approval was granted by the National Research and Ethics Service (Ethical Application Reference 04/Q1604/21; Expiry Date 04/06/2021). Anonymised cases of FFPE lung NSCLC were drawn from the histopathology diagnostic archive at Birmingham Heartlands Hospital and the Cambridge Human Research Tissue Bank, Addenbrooke’s Hospital, Cambridge. For the validation, 40 cases were selected: 20 *EGFR* mutant and 20 *EGFR* wild type, as determined retrospectively by the local standard care test**.** In Birmingham, PCR was the reference standard and this was either the cobas *EGFR* Mutation Test (Roche Molecular Systems Inc.) or the therascreen *EGFR* RGQ PCR Kit (Qiagen), depending upon when the test was carried out. For cases from Cambridge, the reference test was the Ion AmpliSeq™ Cancer Hotspot Panel v2 (Life Technologies). The original H&E sections were examined by a histopathologist and the same tissue area for Idylla™ testing was selected as was originally tested with the reference standard. Idylla™ testing was carried out retrospectively at the John Radcliffe Hospital in Oxford. The general principles and methods for Idylla™ testing have been described previously [10]. Briefly, formalin-fixed paraffin-embedded (FFPE) tumour tissue was either enriched with macro-dissection (resection specimens) from single 5-μm unstained sections on glass slides or unenriched single 5-μm unstained FFPE rolls (small biopsies) were directly submitted for each test. All Idylla™ testing samples met the minimum requirement of tissue with > 10% tumour nuclei content (no minimum tissue dimensions are specified by the manufacturer but a minimum of 2 mm^2^ was used in all cases). FFPE tissue for testing was placed between wetted blotting paper inside an Idylla™ cartridge, which was loaded onto the Idylla™ system for processing. The Idylla™ console software auto-analysed the fluorescent amplification signal to report the presence or absence of a mutation. The presence of a mutation was considered a positive Idylla™ test and wild type was considered negative [10].

The analysis focused on concordance between testing modalities, but also estimated the sensitivity and specificity of the system. Statistical calculations were carried out using standard formulae with Microsoft Excel.

## Results

The raw data from the comparison of Idylla™ and reference testing can be found in Table 2. Twelve cases were biopsies, 28 were resections. There were two cases of squamous cell carcinoma and 38 cases of adenocarcinoma. There was agreement between Idylla™ and standard testing in 37 of the 40 cases giving an overall concordance of 92.5% (95% CI 80.14% to 97.42%) (Table 3). The estimated technical sensitivity given this was 85% (95% CI 63.96% to 94.76%), while the estimated technical specificity was 100% (95% CI 83.89% to 100%).

**Table 2:** The raw data of the comparison between Idylla and reference tests including cobas, therascreen and Ampliseq for *EGFR* testing. Idylla mutations given as reported by the system. The test cannot distinguish between some point mutations (e.g. G719A vs. G719C vs. G719S). Cases in bold represent discordant results

Case no.	Specimen	Reference test	Idylla Results
1	Lung: adenocarcinoma	Exon 21 L858R (therascreen)	Exon 21 L858R
2	Lung: adenocarcinoma	Exon 20 ins (cobas)	Exon 20 ins
3	Lung: adenocarcinoma	Exon 18 G719A/C/S (therascreen)	Exon 18 G719A/C/S
4	Lung: adenocarcinoma	WT (cobas)	WT
5*	Lymph node: SCC	WT (cobas)	WT
6*	Subcarinal tissue: adenocarcinoma	Exon 19 del (therascreen)	Exon 19 del
7	Lung: adenocarcinoma	Exon 21 L858R (therascreen)	Exon 21 L858R
8*	Paratracheal tissue: adenocarcinoma	Exon 19 del (therascreen)	Exon 19 del
9*	Lymph node: adenocarcinoma	Exon 21 L858R (therascreen)	Exon 21 L858R
10*	Pleura: adenocarcinoma	Exon 19 del (therascreen)	Exon 19 del
11	Lung: adenocarcinoma	WT (therascreen)	WT
12	Lung: adenocarcinoma	WT (cobas)	WT
13	Lung: adenocarcinoma	WT (therascreen)	WT
14	Lung: adenocarcinoma	WT (therascreen)	WT
15	Lung: adenocarcinoma	WT (therascreen)	WT
16	Lung: adenocarcinoma	Exon 19 del (therascreen)	Exon 19 del
17	Lung: adenocarcinoma	WT (therascreen)	WT
18	Lung: adenocarcinoma	Exon 21 L858R (therascreen)	Exon 21 L858R
19	Lung: adenocarcinoma	Exon 19 del (cobas)	Exon 19 del
20	Lung: adenocarcinoma	Exon 18 G719A/C/S (therascreen)	Exon 18 G719A/C/S
21	Lung: adenocarcinoma	Exon 21 L858R (therascreen)	Exon 21 L858R
22	Lung: adenocarcinoma	WT (cobas)	WT
23	Lung: adenocarcinoma	WT (therascreen)	WT
24	Lung: adenocarcinoma	WT (therascreen)	WT
25*	Lymph node: SCC	WT (therascreen)	WT
26	Lung: adenocarcinoma	WT (cobas)	WT
27	Lung: adenocarcinoma	Exon 19 del (therascreen)	Exon 19 del
28	Lung: adenocarcinoma	WT (cobas)	WT
29	Lung: adenocarcinoma	Exon 21 L858R (therascreen)	Exon 21 L858R
30	Lung: adenocarcinoma	WT (cobas)	WT
31	Lung: adenocarcinoma	WT (cobas)	WT
32	Lung: adenocarcinoma	WT (cobas)	WT
33	Lung: adenocarcinoma	WT (therascreen)	WT
34	Lung: adenocarcinoma	Exon 19 del (therascreen)	Exon 19 del
35*	Lung: adenocarcinoma	**Exon 20 T790M (AmpliSeq)** **(Incidental exon 18 E709_TdelinsD)**	**WT**
36*	Lung: adenocarcinoma	Exon 19 del (AmpliSeq)	Exon 19 del
37*	Lung: adenocarcinoma	WT (AmpliSeq)	WT
38*	Lung: adenocarcinoma	**Exon 21 L861Q (AmpliSeq)**	**WT**
39*	Lung: adenocarcinoma	**Exon 20 S768I (AmpliSeq)**	**WT**
40*	Lung: adenocarcinoma	WT (AmpliSeq)	WT

*WT*, wild type, *del*, deletion; *in*, insertion; *SCC*, squamous cell carcinoma; *biopsies

**Table 3:** A summary of the results from the comparison of Idylla against reference testing for *EGFR* mutations

	Reference test mutant	Reference test WT	Total
Idylla positive	17	0	17
Idylla negative (WT)	3	20	23
Total	20	20	40

*WT*, wild type

Idylla™ agreed with standard testing in all 34 PCR reference-tested cases (14 of 23 therascreen cases mutant, two of 11 cobas cases mutant), giving 100% concordance (95% CI 89.85% to 100%) with routine (cobas/therascreen) PCR.

The NGS (AmpliSeq) cohort was only six cases (four mutant cases, two wild-type cases), making a subgroup statistical analysis of these of limited value, however, of note was there were three discordant cases. The concordance of Idylla with NGS therefore was only 50%. The three samples were small lung biopsies from lung adenocarcinoma, all of which were called mutant by NGS (see Table 2). The codon changes detected by NGS but designated wild type by Idylla™ in the three cases were exon 20 T790M (low level 4%), exon 20 S768I and exon 21 L861Q (low level 2%). One of the three cases (same case as T790M mutation) also had an incidental exon 18 E709_T710delinsD detected by NGS. This mutation is not covered by the Idylla panel and was considered to be of no clinical importance.

## Discussion

*EGFR* testing is now an integral part of respiratory pathology practice [21] and the clinical demand for urgent (i.e. fast) testing in these patients with a short median survival time is understandably high [22]. The approach to molecular diagnostics for lung cancer differs from centre to centre. In addition to *EGFR*, *ALK*-rearrangement testing is also routine and there are a number of targeted therapies for tumours harbouring the *EML4-ALK* fusion gene [23]. PD-1/PD-L1 and *ROS1* are now also becoming routine [24, 25]. Some centres perform both *EGFR* and *ALK* testing and some centres are using the more widely available *KRAS* testing options to compliment screening (*KRAS* mutations are generally mutually exclusive with *EGFR* and *ALK* mutations, reducing the number of *EGFR/ALK* tests needed). Many laboratories find this easier as lung cancer cases can be batched with other (e.g. colorectal cancer) cases undergoing *KRAS* testing, rather than waiting for sufficient *EGFR* or *ALK* testing samples. Practice is variable, however, and guidelines are not yet fully established for *KRAS* testing [21]. For centres, using the RAS testing approach, the Idylla™ *KRAS* Mutation Test could be integrated into this protocol—although accuracy data for the test in this tissue needs to be generated. *ALK*, PD-1/PD-L1 and *ROS1* testing are not yet available on the Idylla™ platform, however more traditional approaches (immunohistochemistry and/or fluorescence in situ hybridization techniques) already work well [21, 24, 25].

In this study, we evaluated the Idylla™ platform for *EGFR* testing in a representative range of lung cancer histopathology FFPE specimens. This is the first such study to do this for the now commercially available *CE-IVD* approved for clinical use Idylla™ *EGFR* Mutation Test. The results demonstrate high agreement (92.5%) with commonly used molecular tests, although there were three discordant test results. The Idylla™ also showed high estimated specificity (100%) for *EGFR* mutation detection, although this finding is limited by a small sample size and non-random case selection.

There were three discordant cases in this study and this affected the concordance and overall sensitivity of the test. All three cases had *EGFR* mutations that are covered by the Idylla panel. These results were at odds with those reported by others for *EGFR* and Idylla in general and were initially surprising [16, 18]. These results can be explained methodologically. The comparisons were performed on small biopsies with very limited tissue remaining in the block at the time of Idylla™ testing. A pre-Idylla testing H&E was not prepared in order to preserve tissue for the assay, but the original H&E section before NGS testing showed greater than 40% tumour nuclei in all three cases. A follow-up H&E section was cut and stained for the three discordant cases after Idylla testing and these showed that in all three blocks, there were no tumour cells remaining. It is likely that in these cases, there was no tumour DNA present in the samples assayed and that these results do not reflect true discordance. The data could have been improved with a greater number of NGS cases for comparison, but unfortunately, in this small evaluation, there was no funding to cover this. The power of the results could be improved by excluding NGS cases from the statistical analysis altogether; however, it was preferred by the authors to openly publish all data—the inference of the exclusion can still be drawn in the subgroup analysis of the results for PCR alone. This does show the high concordance of Idylla™ with routine PCR.

Although the discordant results were likely due to methodological limitations, some evaluation of the clinical relevance of these (if true discordances) can be speculated. The exon 21 mutation was present at a level of only 2% in the NGS assay and the exon 20 T790M mutation was only detected at 4%; therefore, it is unlikely that cobas or therascreen PCR (the more commonly used assays) would have detected these either. Clinical trial data are limited on the response to initial therapy in patients with these three mutations and so it is not clear if the discordance with Idylla™ is clinically important in these samples anyway [9, 26–30]. Furthermore, T790 is generally more important as a resistance mutation, following EGFR-TKi therapy [31].

The Idylla™ *EGFR* Mutation Test compared with cobas and therascreen PCR assays alone (34 cases) showed 100% concordance. Thus, if NGS cases were excluded from the analysis, this gives an estimated technical sensitivity and specificity of 100%. The main aim of this study was to compare the Idylla with routine PCR because this is probably still the most widely used methodology in Europe (NGS panels are rarely CE approved). The results demonstrate that Idylla is at least as good as the standard care CE–marked testing that is in common use. In light of the fact that the majority of centres are probably still using PCR-based tests, it can be said that the Idylla™ *EGFR* Mutation Test performs equally well as standard care tests for the majority of common and well-characterised lung cancer *EGFR* mutations. The system also offers significant advantages in terms of turn-around times. With the future shift to NGS being extremely likely, some centres may opt for a rapid PCR-based *EGFR* screening test initially in urgent cases and follow this up with NGS later. In this scenario, the Idylla™ would be best suited.

NICE primarily recommends using FFPE tumour biopsy tissue for *EGFR* testing, but acknowledges that often these samples are very small and that testing cytological material may be useful where no tissue is available after the histological assessment has been carried out [5]. A larger study that includes cytological and fresh tissue samples with Idylla™ may therefore be warranted. NICE does not specifically comment on the financial implications of different testing modalities. The Idylla™ *EGFR* Mutation Test costs around £170 per test (Europe-wide average, depending on pricing structure) and is therefore comparable with most conventional *EGFR* PCR assays. In comparison, the cost of NGS gene panels is currently around £300. Therefore, depending on the local arrangements, Idylla™ could potentially reduce costs for some institutions, but for others, this might not be cost effective and the additional financial commitment would need to be balanced against the clinical benefits of reduced turn-around time (which may also be cost saving). A full health economics evaluation of molecular testing in lung cancer could be very helpful.
